# Building the road to a regional zoonoses strategy: A survey of zoonoses programmes in the Americas

**DOI:** 10.1371/journal.pone.0174175

**Published:** 2017-03-23

**Authors:** Melody J. Maxwell, Mary H. Freire de Carvalho, Armando E. Hoet, Marco A. N. Vigilato, Julio C. Pompei, Ottorino Cosivi, Victor J. del Rio Vilas

**Affiliations:** 1 PANAFTOSA (PAHO/WHO), Rio de Janeiro, Brazil; 2 Veterinary Public Health Program, Veterinary Preventive Medicine Department, College of Veterinary Medicine, and Division of Epidemiology, College of Public Health, The Ohio State University, Columbus, Ohio, United States of America; Ross University School of Veterinary Medicine, SAINT KITTS AND NEVIS

## Abstract

**Background:**

In recent years, global public health security has been threatened by zoonotic disease emergence as exemplified by outbreaks of H5N1 and H1N1 influenza, SARS, and most recently Ebola and Zika. Additionally, endemic zoonoses, such as rabies, burden countries year after year, placing demands on limited finances and personnel. To survey the baseline status of the emerging and endemic zoonoses programmes of the Latin American and the Caribbean (LAC) countries, the Pan American Health Organization (PAHO) conducted a survey of priority emerging and endemic zoonoses, countries´ prioritization criteria and methodologies, and suggestions to strengthen countries capacities and regional approaches to zoonoses control.

**Methods:**

A fillable online questionnaire was sent to the zoonoses programme managers of the Ministries of Health (MOH) and Ministries of Agriculture (MAg) of 33 LAC countries from January to April of 2015. The questionnaire comprised 36 single, multiple choice and open-ended questions to inform the objectives of the survey. A descriptive exploratory analysis was completed.

**Results:**

Fifty-four ministries (26 MOH, 25 MAg, and 3 combined responses) in 31 LAC countries responded to the survey. Within the ministries, 22 (85%) MOH, 5 (20%) MAg, and 2 (67%) combined entities indicated they had specialized zoonoses units. For endemic zoonoses, 32 of 54 ministries responded that they conduct formal prioritization exercises, most of them annually (69%). The three priority endemic zoonoses for the MOHs were leptospirosis, rabies, and brucellosis while the three priorities for the MAgs were brucellosis, rabies, and tuberculosis. Diagnosis for rabies and leptospirosis were cited as the capacities most in need of development. The most needed cross-cutting capacity was coordination between stakeholders. For emerging zoonoses, 28 ministries performed formal prioritization exercises. The top prioritization criteria were probability of introduction into the country and impact. The three priority emerging zoonoses for the MOHs were Ebola viral disease, avian influenza, and Chikungunya while for the MAgs were avian influenza, bovine spongiform encephalopathy and West Nile virus disease. Surveillance for avian influenza and Ebola, and diagnosis for BSE were quoted as the capacities most needed. For all zoonoses, the majority of respondents (69%) ranked their relationship with the other Ministry as productive or very productive, and 31% minimally productive. Many countries requested a formal regional network, better regional communication and collaboration, and integrated surveillance.

**Conclusions:**

The survey is the first comprehensive effort to date to inform the status of zoonoses programmes in LAC. The information collected here will be used to develop a regional strategy for zoonoses (both endemic and emerging), increase efforts, advocacy, and promote prompt identification and management of EIDs and improvement of endemic programmes.

## Introduction

In recent years, global public health security has been threatened by zoonotic disease emergence as exemplified by outbreaks of H5N1 and H1N1 influenza, SARS, and most recently Ebola and Zika. In a 2001 risk assessment, it was estimated that 75% of the emerging pathogens were zoonotic [1]. Other studies report that 60 to 70% of emerging infectious diseases (EIDs) events in humans are of animal origin [2, 3]. The rising number of emerging zoonoses may be driven by “modernization of farming practices, particularly in the developing world, habitat destruction, human encroachment and climate change” [3] to support a growing population [4]. This phenomenon holds true in the Latin American and Caribbean (LAC) countries where 70% of the public health emergencies in the Americas reported to the WHO from 2007 to 2008 were classified as zoonoses or communicable diseases common to humans and animals [5].

While emerging zoonoses are often a greater concern to donors and decision-makers, endemic zoonoses have a greater societal impact on neglected populations than emerging diseases [6]. Many endemic zoonoses are part of the group of neglected tropical diseases (NTDs) that “affect mainly poor and marginalized populations in low-resource settings” [7], with their presence reflecting clear inequalities in health. Due to the still significant number of individuals living in extreme poverty in LAC countries (11.5% according to recent World Bank figures [8]) a focus on endemic zoonoses is required. The combined burden of NTDs likely exceeds that from malaria, tuberculosis, and possibly HIV in LAC countries [9].

Despite the importance of emerging and endemic diseases in LAC countries, there are limited national or regional disease burden estimates in the Americas for many zoonoses [9, 10]. Without this information, it is difficult to plan for and adequately provide for disease control and prevention programmes and capacities as per the International Health Regulations (IHR) [11]. The IHR, a binding instrument of international law for all WHO member states, requires countries to strengthen their capacities, such as surveillance, preparedness and response, to address any health risk regardless of its source. Specific for zoonoses, Core Capability 10 addresses capacities for the detection and response to zoonotic events of national or international concern [12].

Given the interaction between environment, animals, and people, a ‘One Health’ (OH) approach has been suggested for effective and efficient control of zoonoses [13]. To that effect, there is a need to map out, across the traditional stakeholders with a role in OH implementation, namely the Ministry of Health and Ministry of Agriculture, the evidence, plans, structures, and processes that would support such efficient control. The likely heterogeneity between Ministries and between countries, about priority diseases, prioritization criteria, and capacity building needs could translate into vulnerabilities in the countries’ zoonoses control efforts. The supranational scope is clear. Zoonoses do not respect countries borders and should be viewed not only as a threat to national health security but also a threat to regional and international health security.

To inform the baseline of priority zoonoses and zoonoses-specific capacities and vulnerabilities in LAC countries, the Zoonoses Unit at the Pan-American Center for Foot-and-Mouth Disease (PANAFTOSA from its acronym in Spanish), a center of the Pan American Health Organization (PAHO), conducted a survey to the Ministries of Health and Agriculture in the region. The study complements the core information regularly captured to inform the status of basic IHR capacities from the countries. To the best of our knowledge, this is the first time that a region-wide survey on emerging and endemic priority zoonoses and related indicators has been conducted in LACs.

## Materials and methods

The zoonoses programme managers of the ministries of health (MOH) and agriculture (MAg) from 33 LAC countries (in South and Central America, Mexico, and the Caribbean) were invited to participate in a survey, which comprised 36 single, multiple choice and open-ended questions, using an online PDF questionnaire created in Adobe Forms Central^®^ (S1 Survey). The questionnaire included a cover sheet explaining the objectives of the survey, clarifying that the identity of individual countries would be protected, and that the data would be presented in an aggregated form for the region. The questionnaire was available in English, Spanish and Portuguese.

The survey collected data on respondent demographics, zoonoses programme resources, priority endemic and emerging zoonotic diseases, prioritization methodologies and criteria, disease specific capacities, current collaborations in zoonotic diseases and suggestions for future technical collaboration. The survey included a glossary to define terms such as ‘emerging,’ ‘endemic’ and ‘prioritization’ (S1 Survey). Five PAHO/WHO experts piloted the survey.

The survey opened in January 2015 and remained open for approximately 5 weeks. Technical support sessions were held during the survey period to assist the completion of the questionnaire. After the official closure of the survey on February 11th, all non-respondents were followed up by email or telephone. The last questionnaire was received April 1, 2015.

The data was analyzed using R open access software (i386 3.1.2) [14] and JMP 11.0.0 [15]. Exploratory data analyses were performed to show response frequencies aggregated at the regional level and compared by ministry (MOH vs. MAg) and by sub-regions (defined as South America, Central America and Mexico, and the Caribbean). Countries’ population, gross domestic product (GDP) and income, as per the World Bank classification [16] were used to inform countries’ comparisons. Results are presented separately for endemic and emerging zoonoses.

The questionnaire requested that respondents be as specific as possible and list genus, species, and subspecies of priority zoonoses. Because not all respondents provided this level of detail, the responses were coded into broad disease categories for further analysis.

Responses to the questions exploring capacity development (three pertaining to endemic zoonoses, and four to emerging) were scored (0 for absence, 1 for presence, and equal fractions adding to 1 if more than two options were possible, e.g. having conducted simulation exercises for each of the three priority emerging zoonoses) and added (to a possible total score of 7), to allow comparisons between countries and sub-regions, in effect generating profiles for capacity building. For countries with both ministries responding, the ministry scores were averaged to calculate the country score. K-Means cluster analysis, using the fpc, pvclust and mclust packages in R, were performed on the 7 emerging and endemic capacity component scores for each country. This exploratory analysis was developed to identify country groupings based on their capacity portfolios to serve as a guide for technical cooperation needs.

## Results

### Resources

Fifty-four ministries (26 MOH, 25 MAg, and 3 combined responses) in 31 (93.9%) of the 33 target LAC countries responded to the survey. Respondents within the Ministries were mostly zoonoses programme managers. Responses from national zoonoses groups with personnel from both ministries were labelled as ‘combined’ for the purposes of this analysis. Responses were received from: Argentina, The Bahamas, Barbados, Bermuda, Brazil, Bolivia, Cayman Islands, Chile, Colombia, Cuba, Dominican Republic, Ecuador, El Salvador, Grenada, Guatemala, Guyana, Haiti, Honduras, Jamaica, Mexico, Nicaragua, Panama, Paraguay, Peru, St. Lucia, St. Vincent and the Grenadines, Suriname, Uruguay, Trinidad and Tobago, The Turks and Caicos Islands, and Venezuela.

Twenty-two (85%) MOH, 5 (20%) MAg, and 2 (67%) combined entities indicated they had specialized zoonoses units. The proportion of South American countries with zoonoses units in their Ministries was smaller (12 ministries, 60%) than that of Central American countries and Mexico (9, 82%), but larger than for Caribbean countries (8 ministries, 35%). The annual budget available for zoonotic diseases programmes at the national level ranged from 69,000 to 6,683,000 USD with a median of 447,000 USD, and was correlated with country GDP (MOH r = .69, p = 0.009; MAg r = .54, p = 0.048). The median number of full-time senior management staff working on the national zoonoses programmes was 1 (range 0–12), 4 for technical staff (range 0–150), and 1.5 for administrative/supporting staff (range 0–57).

### Priorities

Thirty-eight Ministries (70%), 20 (77%) MOHs, 16 (64%) MAgs, and 2 (66%) combined entities, replied that they perform prioritization exercises for endemic diseases or provided a frequency in which the process is performed. When asked how often they prioritize, 22 (58%) prioritize annually, 2 (5%) every 2–3 years, and 5 (13%) every 5–10 years. The remaining 4 (11%) ministries responded that they prioritize *after an outbreak* or at *undefined time to next prioritization*. Five (13%) ministries responded that they prioritize but did not specify a time frame for prioritization.

**Fig 1:**
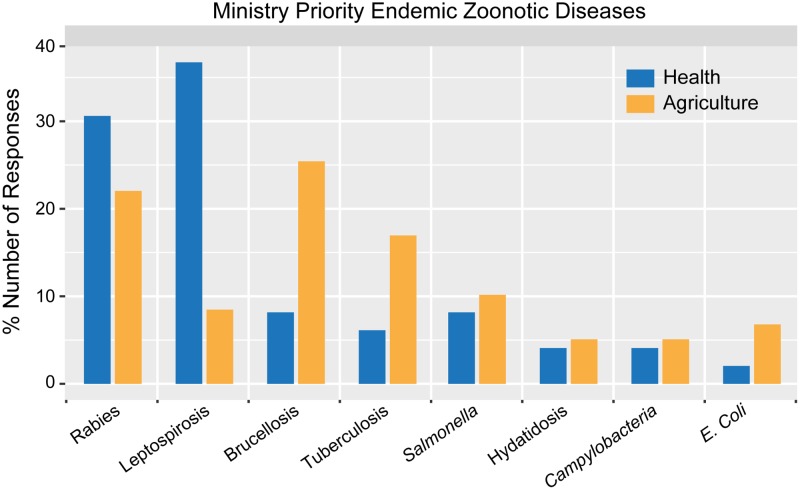
Comparison between the Ministries regarding their top endemic zoonotic disease priorities.

Ministries were asked to list the three priority endemic zoonoses, resulting in 145 responses including 25 unique diseases (Table 1). The disease category “rabies” (including rabies (n = 24), bovine rabies (n = 5) and canine rabies (n = 1)), was reported by 30 ministries (55%), followed by leptospirosis (including leptospirosis (24) and *Leptospira icterohemorragiae* (1)) (25, 46%), brucellosis (including brucellosis (14), bovine brucellosis (4), and caprine brucellosis (1)) (19,35%), tuberculosis (including tuberculosis (7), and bovine tuberculosis (6)) (13, 24%), and *Salmonella* ((*Salmonella* (9), *Salmonella enterica* (1) and *Salmonella* Enteritidis (1)) (11, 20%). The most frequently reported priority endemic zoonoses by MOHs were leptospirosis (18, 69%), rabies (15, 58%), brucellosis (4, 15%), and salmonellosis (4, 15%). For MAgs, the most frequently reported were brucellosis (15, 60%), rabies (13, 52%), and tuberculosis (10, 40%) (Fig 1).

**Table 1:** List of endemic zoonotic diseases and their frequency in regards to their prioritization by the Ministries of Health and Agriculture and combined entities in Latin America and Caribbean countries. First, second, and third refer to the priority level of the zoonotic diseases and total is a sum of all the times that disease was prioritized in such categories.

	Endemic	First	Second	Third	Total
**1**	Rabies	22	2	6	30
**2**	Leptospirosis	4	11	10	25
**3**	Brucellosis	7	6	6	19
**4**	Tuberculosis	2	7	4	13
**5**	*Salmonella*	5	3	3	11
**6**	Hydatidosis	4	0	2	6
**7**	*Campylobacteria*	0	1	4	5
**8**	*Escherichia coli*	1	4	0	5
**9**	Influenza	1	1	2	4
**10**	Chagas	0	3	0	3
**11**	Leishmaniasis	2	1	0	3
**12**	Venezuelan Equine Encephalitis	0	2	0	2
**13**	*Trichinella spiralis*	0	0	2	2
**14**	Hantavirus	0	0	2	2
**15**	Plague	0	2	0	2
**16**	Anthrax	0	1	1	2
**17**	Chikungunya	2	0	0	2
**18**	Equine Encephalitis	0	1	0	1
**19**	Dengue	1	0	0	1
**20**	Helminths	0	0	1	1
**21**	Food Borne Illness	0	0	1	1
**22**	Toxoplasmosis	0	1	0	1
**23**	Fasciolosis	0	1	0	1
**24**	Erysipelas	0	1	0	1
**25**	*Burkholderia mallei*	0	1	0	1
**26**	Other^1^	0	0	1	1

^1^The disease category ‘Other’ includes snake bite, which is not zoonotic.

The number of criteria used by the ministries to prioritize endemic zoonoses ranged from 0–14, with 50 ministries (93%) reporting the use of at least one criterion. On average, ministries used 4.72 criteria per zoonosis. The criteria most frequently reported as used by the MOHs in their prioritization exercises were, in order of frequency, human disease incidence, human disease severity, human disease mortality, and human disease prevalence. For MAgs the criteria were economic impact, animal disease prevalence, human disease incidence, and animal disease incidence. Animal welfare, public opinion and DALYs were the criteria least frequently used by both ministries in their prioritizations. Respondents were asked to describe the methodologies used to aggregate the multiple impacts assessments across criteria. Most ministries reported using *expert opinion* (19, 59%), eight (25%) used *multi-criteria decision techniques*, five (16%) used *epidemiology values*, and three (9%) reported other approaches (*producer demands*, *an analysis of indicators*, and *meetings regarding control and prevention*).

For emerging zoonoses, 31 (57%) ministries, 16 (64%) MAgs, 14 (54%) MOHs, and 1 (33%) combined entity), completed formal prioritization exercises. Each ministry was asked to list their three emerging priority zoonoses, resulting in 130 responses including 25 diseases (Table 2). The most frequently reported emerging zoonoses were avian influenza (AI) (avian influenza (21), H5N1 (3), H7N9 (1), and highly pathogenic avian influenza (6)) (31 ministries, 57%), Ebola virus disease (EVD) (19, 35%), and Bovine Spongiform Encephalopathy (BSE) (15, 28%). The most frequently reported emerging zoonoses by MOHs were EVD (14, 54%), AI (11, 42%), and Chikungunya (8, 31%). For MAgs, the most frequently reported were AI (18, 72%), BSE (10, 40%), and West Nile virus (WNV) and rabies (7, 28% each).

**Table 2:** List of emerging zoonotic diseases and their frequency in regards to their prioritization by the Ministries of Health and Agriculture and combined entities in Latin American and Caribbean countries. First, second, and third refer to the priority level of the zoonotic diseases and total is a sum of all the times that disease was prioritized in such categories.

	Emerging	First	Second	Third	Total
**1**	Avian Influenza	18	9	4	31
**2**	Ebola Viral Disease	14	2	3	19
**3**	Bovine Spongiform Encephalitis	4	6	5	15
**4**	Chikungunya	1	10	1	12
**5**	West Nile Virus	1	5	5	11
**6**	Rabies	4	3	2	9
**7**	Hantavirus	0	1	3	4
**8**	Dengue	3	0	0	3
**9**	Equine Encephalitis	0	0	2	2
**10**	*Trichinella spiralis*	0	2	0	2
**11**	MERS-CoV^1^	0	1	1	2
**12**	Lyme Disease	0	1	1	2
**13**	Creutzfeldt Jacob	0	1	1	2
**14**	Leptospirosis	1	0	1	2
**15**	Leishmaniasis	1	0	1	2
**16**	Influenza	1	0	1	2
**17**	Saint Louis Encephalitis Virus	0	0	1	1
**18**	Food Borne Illness	0	0	1	1
**19**	Echinococcus	0	0	1	1
**20**	Anthrax	0	0	1	1
**21**	Taenia	0	1	0	1
**22**	Screwworm	0	1	0	1
**23**	*Salmonella*	0	1	0	1
**24**	*Escherichia coli*	1	0	0	1
**25**	Brucellosis	1	0	0	1
**26**	Other^2^	0	3	1	4

^1^Middle East Respiratory Syndrome Coronavirus

^2^The disease category ‘Other’ includes classical swine fever, foot-and-mouth disease, Newcastle disease, and antimicrobial resistance.

Of the 31 ministries that completed prioritization exercises for emerging zoonoses, 30 considered the probability of introduction of the emerging condition in their countries as a criterion in their prioritizations, and 29 the impact of such introduction. Impact on public health, society, the economy, and the environment were the most frequently reported by both ministries. Other impacts used by both ministries included the impact on tourism, animal health and agriculture, international relations, and food security. Ministries also considered criteria such as international sanitary regulations and the probability of rapid transmission in their prioritization of emerging zoonoses. Only 18 (33%) of the ministries (6 MAg (24%), and 12 MOH (46%)) considered equity as a criterion in their prioritization and allocation of resources for the control of zoonoses, whether endemic or emerging.

**Fig 2:**
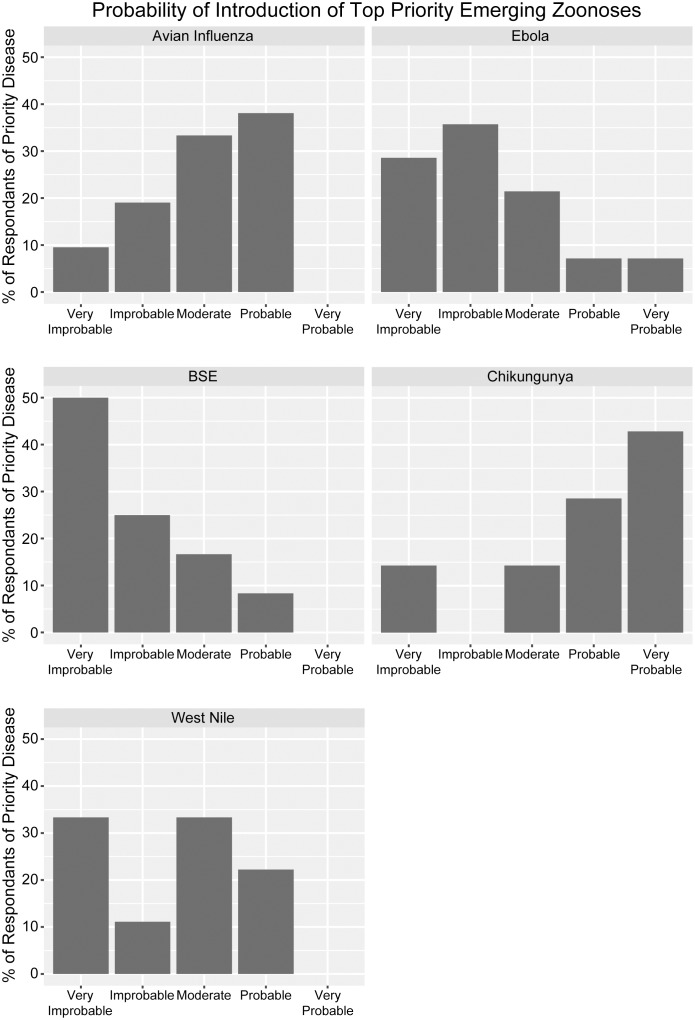
Probability of introduction for the top emerging zoonotic diseases in Latin American and Caribbean countries. Probabilities were selected by the Ministries of Health, Agriculture, and combined entities (*very improbable* (probability < 0.20), *improbable* (0.20–0.40), *moderate* (0.41–0.60), *probable* (0.61–0.80), and *very probable* (0.81–1.00).

Ministries were asked to select from a range (from very probable (0.81–1.00) to very unlikely (<0.20)) the probability of introduction of their priority emerging zoonoses into their countries. Approximately 85% of the ministries reported moderate to very probable the introduction of Chikungunya; 71% moderate to probable the introduction of AI; 56% moderate to probable the introduction of WNV; 36% moderate to very probable the introduction of EVD; and 25% moderate to probable the introduction of BSE. Chikungunya and EVD were the only two conditions reported as of very probable introduction by 43% and 7% of respondents, respectively (Fig 2). While ministries were asked for the timeline regarding the probability of introduction, the majority did not know or chose not to answer this question.

### Analysis of capabilities

**Fig 3:**
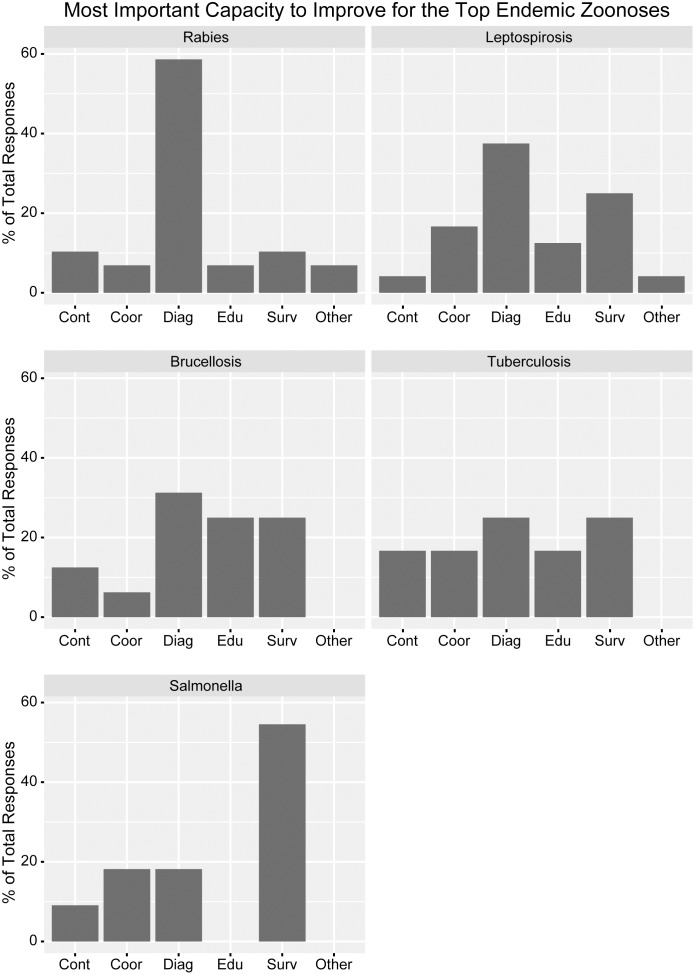
Top capacities that require improvement for each of the top five endemic priority zoonoses. The Ministries of Health and Agriculture and combined entities of Latin American and Caribbean countries listed capacities to improve primarily within five categories (control, *cont*; coordination, *coor*; diagnosis, *diag*; education, *edu*; and surveillance, *surv*). Responses that fell outside these categories were categorized as *other*. Percentage represents total respondents received per zoonosis.

**Fig 4:**
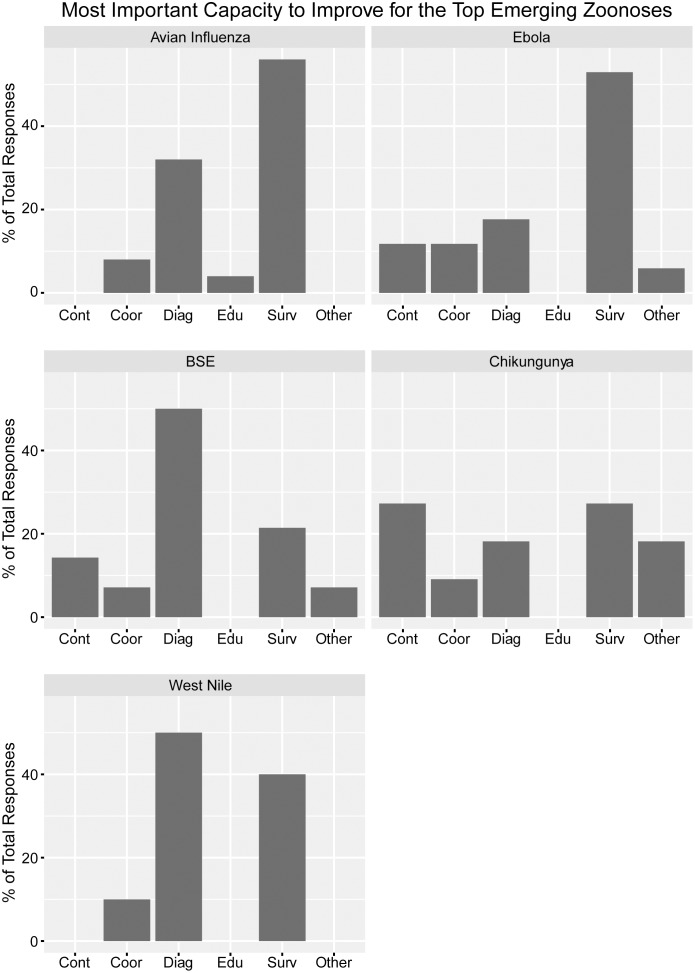
Top capacities that require improvement for each of the top five emerging priority zoonoses. The Ministries of Health and Agriculture and combined entities of Latin American and Caribbean countries listed capacities to improve primarily within five categories (control, *cont*; coordination, *coor*; diagnosis, *diag*; education, *edu*; and surveillance, *surv*). Responses that fell outside these categories were categorized as *other*. Percentage represents total respondents received per zoonosis.

Surveillance for emerging conditions and diagnostics for endemic conditions were reported as the capabilities most in need of improvement. In more detail, most ministries cited diagnosis and laboratory capabilities as the most important disease-specific capability requiring improvement for rabies, leptospirosis, and brucellosis (Fig 3). Surveillance for AI and EVD, and diagnosis for BSE and WNV were the most demanded capabilities for emerging zoonoses (Fig 4). Forty-three respondents reported that they are currently conducting syndromic surveillance on endemic and/or emerging conditions (MAg 21 (84%); and MOH 22 (88%)).

**Fig 5:**
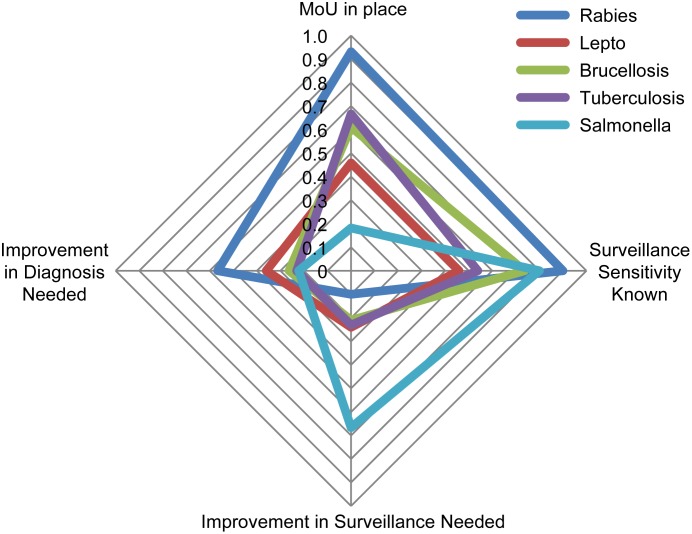
Comparison of the top five priority endemic zoonoses for Latin American and Caribbean countries.

**Fig 6:**
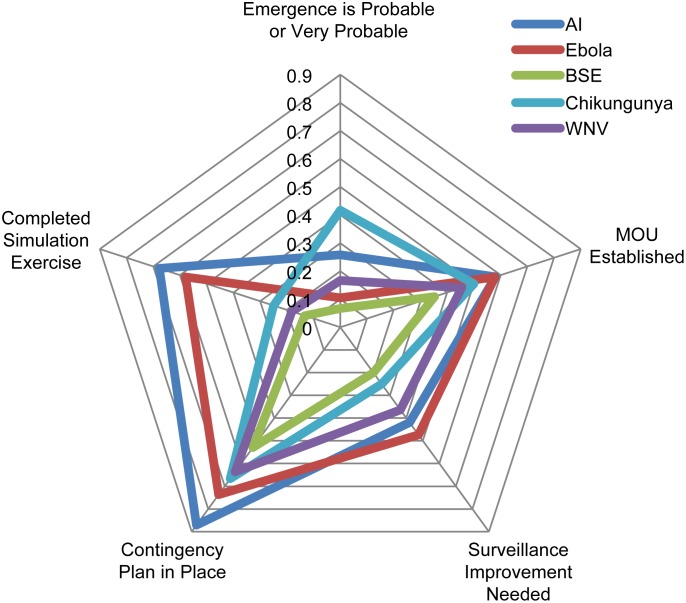
Comparison of the top five priority emerging zoonoses of Latin American and Caribbean countries.

Focusing on the priority endemic zoonoses, 93% of respondents prioritizing rabies replied that they had a current formal agreement or Memorandum of Understanding (MoU) with other government ministries for the coordinated prediction, prevention, detection, and intervention of rabies, 67% for tuberculosis, 61% for Brucellosis, 46% for leptospirosis, and 18% for salmonellosis (Fig 5). Ninety percent of the ministries replied that they knew the sensitivity of their rabies surveillance systems, 80% of their salmonella systems, 74% in the case of brucellosis, 54% of tuberculosis, and 44% for leptospirosis (Fig 5). For the priority emerging zoonoses, capacities against AI appear best developed with 58% of respondents prioritizing AI stating that they had a MoU in place, 68% completed a simulation exercise in the last five years, and 87% had up-to-date contingency plans (Fig 6).

**Fig 7:**
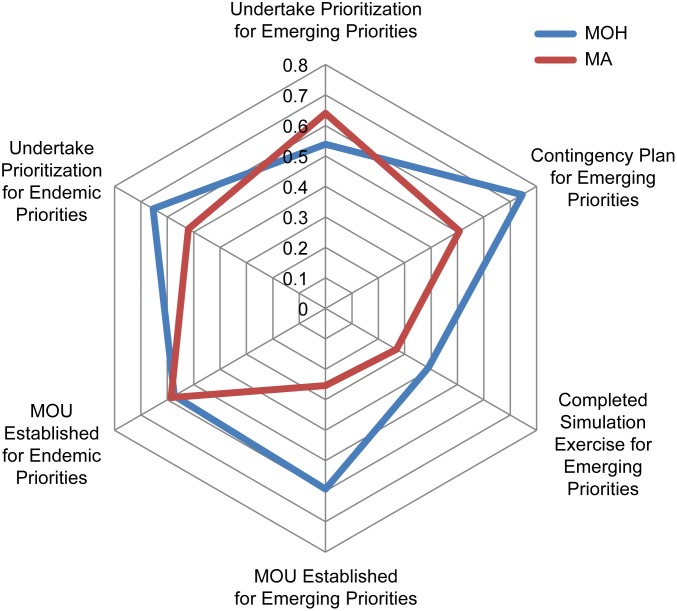
Comparison of activities completed by the Ministries of Health and Agriculture regarding endemic and emerging zoonoses.

**Fig 8:**
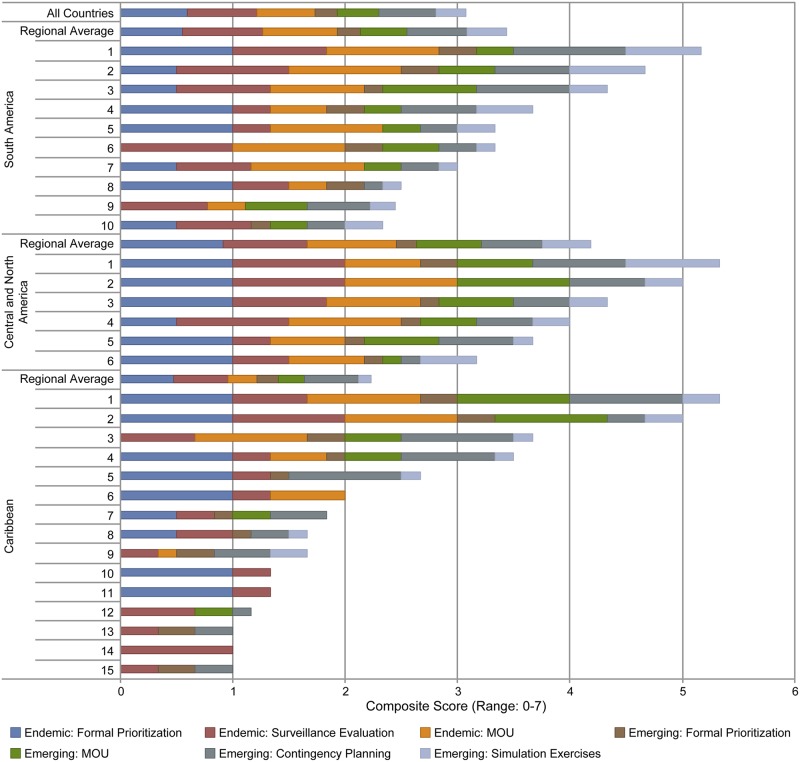
Development of capacities against emerging and endemic zoonoses per country. Seven capacities were assessed for each country. Countries are grouped by sub-region (South America, Central and North America, and the Caribbean). The first bar shows the average capacity development for all countries that responded to the survey (31). Regional averages are also shown for each region to allow comparisons between countries.

In general, MOHs showed greater capacity development than MAgs (Fig 7). Countries’ capacities’ scores, aggregated by sub-region show lower overall scores for Caribbean countries (Fig 8). Of a maximum score of 7, the mean capacity score was 4.25, 3.48 and 2.28 in Central-America and Mexico, South America and Caribbean sub-regions, respectively. While the hierarchical cluster analysis was exploratory in nature, whether or not a country prioritized endemic disease was a primary driver of clustering.

Thirty-seven ministries (69%) reported their relationship with the other ministries involved in the control of zoonoses in their countries as productive or very productive, and 17 (31%) as minimally productive with few attempts of coordination. No ministry reported the absence of any coordination with other ministries. Only 24 ministries (44%) have a formal written agreement with universities, 13 (24%) with non-governmental organization, 18 (33%) with the private sector, and 10 (19%) with other organizations including scientific groups, the central government, regional authorities, emergency committees, and the community. Ministries were also asked to produce suggestions of activities to improve coordination and collaboration with the other ministries for the control of zoonoses. Fourteen ministries (26%) suggested the formalization of collaboration, such as through an MOU, regular meetings (10, 19%), data sharing (10, 19%), and joint planning (7, 13%). Additional suggestions included forming an interagency group, and joint capacity training.

The majority of respondents (48, 89%) allocated very high or high value to a regular report on the occurrence of zoonoses in the Americas region with the information gathered in this report. When asked about the most critical development that the American region requires for the control of zoonoses, 21 (39%) of ministries requested a formal network, nineteen (35%) recommended better regional communication and collaboration, and nineteen (35%) responded with integrated surveillance. Other suggestions included integrated vector control, training of veterinarians and public health professionals, an integrated regional task force for emergencies, effective implementation of International Health Regulations, formal agreements between countries, strengthening current networks such as Caribvet, standardization of current zoonoses programmes, and a regular report on emerging and endemic zoonoses.

## Discussion

The present study reports the results of the first survey to Ministries of Health and Agriculture across the American region on priority zoonoses and capacities. The response rate to the survey, 31 countries out of 33, evenly distributed across MOH and MAg, is the most significant output of this study, and correlates with their engagement towards building more integrated and regional-wide mechanisms and processes for the control of zoonoses.

Twenty-nine Ministries (54% of all respondents) replied that they had specialized zoonoses units. Of those, only 20% of MAgs replied they had zoonoses units, compared to 85% of MOHs. This difference may stem from the prominent focus of MAg in the region towards animal health, production, and trade. This observation is supported by the selection of *economic impact* as the first prioritization criterion by MAgs, and in the subsequent identification of AI and BSE as their top priority emerging zoonoses, as the presence of either disease would lead to severe trade consequences.

The top criteria for prioritization of endemic zoonoses *were human incidence*, *economic impact*, *animal prevalence*, and *human severity*. Ministries did not frequently report the use of public opinion, animal welfare or DALYs as prioritizing criteria. This could be due to the difficulty of measuring them, integrating them with the other criteria, and in the case of public opinion and animal welfare to an implicit reduced relevance relative to other criteria deemed as more important. Public opinion however is a frequently used criterion in decision frameworks involving public goods or services, with multiple impacts and stakeholders, and shaped by severe uncertainty [17]. This decision context appears to apply to many of the zoonoses prioritized by the Ministries. Similarly, DALYs did not rank prominently, probably due to the lack of data to consistently inform such a composite metric for many of the neglected zoonoses. This argument supports earlier reports on the shortage of studies regarding the burden of many zoonoses in the Region [9]. Recent publications have started to fill this gap, including an estimate of intermediate burdens of foodborne DALYs, many of which are zoonotic, for the LAC region [10]. An FAO/WHO initiative using an expert-based multicriteria ranking tool for assessing foodborne parasitic diseases noted a deficit of data for Central American countries, but the same was not noted for South American countries [18].

When asked about emerging zoonoses prioritization, most Ministries replied that they considered a range of impacts and the probability of introduction in their prioritizations of emerging conditions (Fig 2). However, very few respondents assessed the probability of introduction within a defined timescale (e.g. within 6 months, 1 year). Future surveys will have to explore this further as well as the methods Ministries used to derive the combined impact on the multiple criteria, especially given the divergent scales of the impacts, e.g. public health vs. economic impact. Unsurprisingly, emerging zoonoses priorities were influenced by the timing of the survey delivery. The survey was delivered during early 2015, just after the peak of the EVD outbreak in Western Africa. Its influence in the responses was clear as 19 Ministries reported EVD as a priority, even when the reported probability of introduction was very low (Fig 2). This shows the larger weight given to the possible impact of EVD over its unlikely introduction.

Despite the widespread recognition of the disproportionate impact of zoonoses on marginalized populations [7], only 33% of Ministries stated that they consider equity issues in their definition of priorities and allocation of resources against emerging and/or endemic zoonoses. Numbers were particularly low for MAgs (only 23% considered equity), which suggests significant opportunity for MAgs to enhance their role in the wellbeing of the farming community, by reaching out to marginalized producers and their families at higher risk of occupational exposure to a number of zoonoses. This would compensate for the concentration of MAgs competencies focused on the importance of the livestock sector as a national product, and enhance the current unclear role of veterinary governance on poverty reduction [19].

Disease prioritization is a compensatory exercise and formal techniques are required to handle the multiple trade-offs and methodological problems that regularly arise in the process [20, 21]. Improvement and standardization of prioritization methods across the Region are possible as only eight ministries replied that they used multi-criteria-decision techniques for the aggregation of impacts across prioritization criteria. The majority of respondents used expert opinion (59%) or other approaches. While 70% of ministries replied that they perform prioritization exercises for endemic diseases or gave a time frame for prioritization, our results fall short of the proportion of IHR Core Capacity Implementation respondents survey who reported having a list of priority zoonoses (94%) with case definitions [22]. This difference in proportion may be due to a difference in respondents or it may also denote the proportion of ad-hoc prioritization exercises which occur in the region. Future studies will have to explore the robustness of these ad-hoc approaches.

### Limitations

Follow-up of some answers merit study. Ninety percent of respondents (in the case of rabies) stated that they knew the sensitivity of their surveillance. This appears in contrast to results from a review of emerging zoonoses surveillance systems that reported that surveillance evaluation was very rare [23]. We would expect similar results for endemic zoonoses. Furthermore, it is known to the authors that there are no or few countries in the Region with the capacity of evaluating their rabies surveillance and robustly estimating its sensitivity.

Although Ministries were requested to be as specific as possible in naming their priority zoonoses, a number of them only informed generic names, e.g. “rabies”. Other Ministries, however, produced specific responses, e.g. dog-mediated rabies. Given this heterogeneity, answers were pooled under disease categories. This required some judgment by the authors that led to some loss of information (e.g. pooling answers referring to canine or sylvatic rabies under the rabies category combined two different epidemiological entities that warrant different controls by different Ministries). A feasible solution for future surveys is the incorporation of compulsory fields in the questionnaire to capture all the information up to serotypes or variants.

Additionally, a number of ministries included non-zoonotic vectorborne diseases, e.g. Chikungunya or dengue, in their responses, possibly because these diseases are integrated within the zoonoses programmes. On the other hand, foodborne zoonoses may not be integrated into the traditional zoonoses programmes. The ministries were asked to consider foodborne zoonoses when responding to the survey, but, due to potentially differing foodborne and traditional zoonoses programmes, the survey may have failed to collect complete information regarding foodborne zoonoses.

Although most Ministries were able to provide reasonable risk pathways for the introduction of their priority emerging zoonoses, few Ministries considered them in their risk analyses. This could be due to the methodological challenges of quantifying the pathways’ relative risks and their incorporation in a comprehensive risk assessment. Future work needs to focus on how often and thoroughly respondents assess their risk pathways, and what methodologies they use to combine probability, impact, and vulnerability for each pathway.

The survey explicitly stated that the unit of interest was the national or federal level. Given the widespread decentralization of health and agriculture responsibilities towards state and municipalities across the Region, compilation of information from lower administrative levels would have required expensive consultations within countries. Despite this explicit request, it is possible that some countries provided countrywide information as shown by the large upper range value of some responses (e.g. number of staff employed within zoonoses programmes). Additionally, the required simplification of a regional survey such as this obviously fails to capture the heterogeneity within countries, as pooled country-level responses may not reflect the priorities of states and provinces.

### Capacities

Our results allow the identification of regional vulnerabilities (both disease-specific and across diseases), as well as of countries lagging behind the region’s benchmark. Although not presented here, individual countries and Ministries can be profiled to help identify potential vulnerable hot-spots that warrant technical cooperation from neighboring countries and international agencies. Regional and country-specific profiles allow comparisons with the countries responses to the annual IHR Core Capacity Implementation Survey. In 2013, 97% of the countries within the Americas region reported coordination within government for the detection and response to zoonotic events, and 97% had functional mechanisms for collaboration between animal and human health units such as intersectoral surveillance units and laboratories [22]. These compare with the results of the present survey where Ministries were asked to rank the relationship with their counterparts. None of the Ministries ranked their collaboration with the other Ministry as non-existent. In fact, the majority of Ministries ranked the collaboration as very or overall productive (69%). Yet, many Ministries suggested improvements through regular meetings, formalized MoUs, and data sharing. The latter entails a highly evolved relationship and well developed processes between parties and is uncommon, as reported by Vrbova et al. [23] who found that only 19% of the surveillance systems for emerging zoonoses considered both human and animal data. On a related topic, albeit less demanding of sophisticated structures and processes to support useful data sharing, the 2013 IHR Core Capacity Implementation survey reported that 80% of countries in the Americas had a timely and systematic exchange of information between the animal and human sectors [22]. Because so few MAgs have a dedicated zoonoses group (20%), the collection and sharing of data beneficial to both parties is likely to remain a challenge. For emerging zoonoses, given the low proportion of respondents that replied that they had an MoU with their counterpart (Fig 6), opportunities to improve this specific capacity appear possible. This is likely to hold true only for those zoonoses that have both a significant public health and an economic impact (e.g. BSE, AI). For other conditions, MoH may not perceive much benefit in establishing an MoU with MAgs, due to the little involvement of the latter in the control of zoonoses, e.g leptospirosis.

In the case of simulation exercises, their frequency is dependent on the reported/perceived probability of introduction of the emerging zoonosis of interest. On this note, 80% of the respondents considered the probability of introduction of BSE very improbable or improbable. This could explain the very low uptake of simulation exercises by the Ministries. In the case of Chikungunya, although the reported probability of introduction was high, the proportion of Ministries that conducted simulation exercises was low. A possible explanation is that Chikungunya response plans could be embedded within the response plans of other arboviruses. Finally, although the majority of countries (64%) classified the probability of EVD introduction as very improbable or improbable, the proportion of countries that comprehensively prepared for it was large (Fig 6), indicating that the impact of the disease weighed relatively more in their decisions.

Efforts for the control of high-impact diseases (e.g. AI) have highlighted the need for intersectoral cooperation, and shown that success is possible when functional collaborations between national stakeholders are established [24]. Despite extensive recognition of the need for a wide stakeholder base for the control of zoonoses, only 24% of the Ministries of this Region had a formal written agreement with non-governmental organizations, 33% with the private sector, and 19% with other organizations including scientific groups, the central government, regional authorities, emergency committees, and the community.

While there exists a number of supra-national initiatives targeted at, mostly endemic, specific zoonoses, e.g. canine rabies and cystic hydatidosis [25, 26], the Region does not possess an overarching strategy to, among other objectives, realize the potential benefits of integrating shared resources across vertical zoonoses programmes. Such integration would lead to improved control and eradication programmes as well as conservation of resources. Numerous activities, some of them captured by our survey, e.g. a regional report on zoonoses and the development of regional mechanisms to manage emerging zoonoses, would be better delivered through such an overarching strategy. The evidence captured here will be used to inform PAHO’s regional zoonoses strategy, which will, as part of a One Health vision, constitute an important mechanism to support the organization’s commitment towards the Development Goals [27]. This was the theme of the recent 17^th^ Inter-Ministerial Meeting of Health and Agriculture Ministries (RIMSA in Spanish) held in Asuncion, Paraguay in July 2016 [28].

## Conclusion

The current survey provides evidence of the region’s priority zoonoses, and contributes a baseline of basic zoonoses programme indicators on which to target technical cooperation initiatives. A number of improvements appeared evident: i) standardization of prioritization approaches, surveillance definitions and evaluation processes to support comparisons, ii) greater communication and coordination between countries, and iii) a platform to inform zoonoses occurrence in the region and the status of the region´s capacities. All these improvements would lead to a more accessible pool of comparable evidence to inform prioritization exercises.

## Supporting information

S1 Survey Zoonotic diseases survey in English. The survey questionnaire was comprised of 36 single, multiple choice and open-ended questions.
(PDF)
