# Supplementary material for: Building the road to a regional zoonoses strategy: A survey of zoonoses programmes in the Americas
Source: PLoS One. 2017 Mar 23;12(3):e0174175. doi: 10.1371/journal.pone.0174175 (PMC5363932; doi:10.1371/journal.pone.0174175)
Supplement: S1 Survey — The survey questionnaire was comprised of 36 single, multiple choice and open-ended questions. (PDF) [file pone.0174175.s001.pdf]

Zoonotic Diseases Survey

Given the relevance of zoonoses in the world and to the Americas region, the zoonoses unit within the Pan-American Center for Foot-and-Mouth Disease (PANAFTOSA) of the Pan-American Health Organization (PAHO) is conducting a survey of national zoonotic disease organizations within the Americas region. This survey will help to determine the zoonotic diseases of importance to the region (both EMERGING and ENDEMIC), learn more about active zoonoses programs, and determine the current needs of member states. This information will also be used to prepare a regional assessment regarding the regional needs to emerging zoonotic disease threats, further characterize the regional situation and identify benchmark practices. We hope to strategically prioritize the technical cooperation of PANAFTOSA. Your participation in this survey will greatly assist these efforts.

Survey instructions

1. The survey is made of 36 questions. You can complete the survey online or download it as a PDF file. If you complete the survey as PDF, you can save any progress and continue at a later time. Once you complete the survey online or the PDF file, please click "**submit**" at the end of the form (after the Glossary).
2. The survey targets endemic and emerging zoonoses in your country.
3. Please remember to consider the vast variety of zoonoses including those transmitted by food, wildlife, etc. This is critical so your responses clearly reflect the priorities of your country.
4. **A glossary of terms is located at the end of the form.**
5. The survey comprises different sections that may require the input of more than one person. However, we advise that one person coordinate the form's completion and submission.
6. The time estimated for completing the survey is around one hour
7. We kindly ask you to return the form by **February 13, 2015**
8. No countries will be identified; all results will be presented in an aggregated format and the data will be kept confidential.

The survey presents an ideal situation and aims to identify the regional position against the highest standards for zoonoses surveillance and control. This is very difficult to attain in practice. As a result, you may not be able to respond to some of the questions. This is intentional. We thank you for your understanding.

If you have any doubts or questions about the survey or its completion, please contact us at the following email addresses: Victor Del Rio (vdelrio@paho.org) and Melody Maxwell (mmaxwell@paho.org).

Thank you for your time.

PANAFTOSA Zoonoses Unit

Section I Demographics

1. Country

____

2. Organization/Affiliation (e.g. Ministry of Health; Ministry of Agriculture)

____

3. Department within Organization

____

4. Name of the Person Coordinating the Completion of the Whole Form

____

5. Position within the Organization

____

6. Email of the Person Coordinating the Completion of the Whole Form

____

7. Telephone Number (including the country code and city code) of the Person Coordinating the Completion of the Whole Form

____

Section II Resources

8. Does your Ministry have a specific zoonoses group/department/division?

- ○ Yes, we have one specific unit for zoonotic diseases
- ○ Yes, but we are separated further (e.g. vector-borne diseases, or food-borne zoonoses, etc)
- ○ No
- ○ N/A, please specify why: ____

9. Please provide the budget available for zoonotic diseases (even if you don't have a specific zoonoses unit) in 2014 (in thousands of US $). We recognize that in many countries health-related funds are decentralized. We are NOT asking for these. We only ask for those directly administered at the national level within the country's Ministry. Please provide details in thousands of US $ for:

9.1. Personnel (e.g. $700,000):

____

9.2. Program Activities (e.g. surveillance, control, etc) (e.g. $120,000):

____

9.3. Others (e.g. $16,000):

____

9.4. N/A, please describe why:

____

10. How many full time equivalent personnel work on zoonotic diseases for the following categories in your ministry? For example, if there are three full time technical staff and one half time technical staff, enter 3.5.

10.1. Senior management (e.g. zoonoses coordination)	10.2. Technical staff (non-managerial role)	10.3 Administrative staff
____	____	____

Section III Priorities

Questions 11 to 19 refer specifically to ENDEMIC zoonoses (that is, those already present in your country)

We understand that there are different levels at which disease prioritization can be performed (for all diseases, for communicable diseases only, for zoonoses only). We only want to know if zoonoses are prioritized, either as part of a larger prioritization exercise or not, and which zoonoses are most important to your country. We also understand that ideally the criteria for prioritization should be the same for all diseases, however, we provide options for each zoonosis in case different approaches were followed.

11. Does your Ministry conduct a **formal,** i.e .planned, collaborative and representative, prioritization exercise leading to the identification and ranking of the endemic zoonoses most important to your country?

- ○ Yes
- ○ No
- ○ Other (please describe): ____

12. Based on the formal prioritization, as in question 11, please list your top three **endemic** zoonoses (please consider foodborne, vector-borne, wildlife, etc) from most important to least important reflecting the **current** priorities for your **organization**. If you answered "No" to question 11, please provide the three **endemic** zoonoses that you consider priority based on your experience. Please be as specific as possible and list genus, species, and subspecies if possible (e.g. *Escherichia coli* O157:H7).

12.1. **Top priority** endemic zoonosis

[ ]

12.2. **Second priority** endemic zoonosis

[ ]

12.3. **Third priority** endemic zoonosis

[ ]

13.1. Either based on your experience, or from the formal prioritization exercise conducted by your Ministry, as per your answer to question 11, please describe what criteria you used to rank the **first** priority endemic zoonosis in your country (as in question 12.1). Please tick all the criteria that apply

- ☐ Human prevalence
- ☐ Human incidence
- ☐ Severity in humans
- ☐ Human mortality
- ☐ DALYs
- ☐ Society impact
- ☐ Animal/Herd prevalence
- ☐ Animal/Herd Incidence
- ☐ Animal mortality
- ☐ Animal welfare
- ☐ Public opinion
- ☐ Economic impact
- ☐ Other (please describe) [ ]

13.2. Either based on your experience, or from the formal prioritization exercise conducted by your Ministry, as per your answer to question 11, please describe what criteria you used to rank the **second** priority endemic zoonosis in your country (as in question 12.2). Please tick all the criteria that apply

- ☐ Human prevalence
- ☐ Human incidence
- ☐ Severity in humans
- ☐ Human mortality
- ☐ DALYs
- ☐ Society impact
- ☐ Animal/Herd prevalence
- ☐ Animal/Herd Incidence
- ☐ Animal mortality
- ☐ Animal welfare
- ☐ Public opinion
- ☐ Economic impact
- ☐ Other (please describe) [ ]

13.3. Either based on your experience, or from the formal prioritization exercise conducted by your Ministry, as per your answer to question 11, please describe what criteria you used to rank the **third** priority endemic zoonosis in your country (as in question 12.3). Please tick all the criteria that apply

- ☐ Human prevalence
- ☐ Human incidence
- ☐ Severity in humans
- ☐ Human mortality
- ☐ DALYs
- ☐ Society impact
- ☐ Animal/Herd prevalence
- ☐ Animal/Herd Incidence
- ☐ Animal mortality
- ☐ Animal welfare
- ☐ Public opinion
- ☐ Economic impact
- ☐ Other (please describe) [ ]

14. If your Ministry used more than one criteria to prioritize the endemic zoonoses, please describe how (i.e. what method) your Ministry combined the multiple criteria (e.g. subject matter expert consensus, multi-criteria-decision-analysis techniques). Otherwise, write "NA"

[ ]

15. How often does your organization prioritize endemic zoonoses?. If your organization does not formally prioritize endemic zoonoses, please select "N/A".

- ○ Every year
- ○ Every 2 years
- ○ Every 3 years
- ○ N/A
- ○ Other please describe: [ ]

16. For the three most important endemic zoonoses, do you evaluate your surveillance systems sensitivity (i.e. the proportion of **all cases in the population**, human for the Ministry of Health, animal for the Ministry of Agriculture, detected by the surveillance systems)?

16.1. For the most important endemic zoonosis

- ○ Yes
- ○ No
- ○ Other (describe) [ ]

16.2. For the second most important endemic zoonosis

- ○ Yes
- ○ No
- ○ Other (describe) [ ]

16.3. For the third most important endemic zoonosis

- ○ Yes
- ○ No
- ○ Other (describe) [ ]

17. Which **disease-specific** capacity (e.g. disease surveillance, laboratory diagnostic facilities, coordination of stakeholders) are you most interested in developing further in your country for your priority **endemic** zoonoses.

17.1 For your top endemic zoonosis (just the most important capacity)

[ ]

17.2 For your second most important endemic zoonosis (just the most important capacity)

[ ]

17.3. For the third most important endemic zoonosis (just the most important capacity)

[ ]

18. Please describe the most critical cross-cutting capacity (i.e. **not disease specific**) to develop further in your country to enhance the management of endemic zoonoses (e.g. better integration with other stakeholders, etc)

[ ]

19. Do you have a current Memorandum of Understanding or other form of formal agreement with other government ministries (ie Ministry of Agriculture or Ministry of Health) for the coordinated prediction, prevention, detection and intervention of the three most important **endemic** zoonoses? Please tick all that apply.

- ☐ For the top priority endemic zoonosis
- ☐ For the second priority endemic zoonosis
- ☐ For the third priority endemic zoonosis
- ☐ Other (please describe) [ ]

Questions 20 to 26 refer to EMERGING/EXOTIC zoonoses (that is, those not currently present in your country)

We understand that you may have prioritized jointly endemic and emerging zoonoses. However, other countries may have followed a different approach. This is why emerging zoonoses, and their prioritization criteria are asked separately.

20. Does your Ministry conduct a **formal,** i.e .planned, collaborative and representative, prioritization exercise leading to the identification and ranking of the **emerging** zoonoses most important to your country?

- ○ Yes
- ○ No
- ○ Other (please describe) [ ]

20.1 Does the prioritization of emerging/exotic zoonoses consider the following criteria? Tick all that apply. If you answered "No" to question 20, please select "NA".

- ☐ probability of introduction to your country
- ☐ impact
- ☐ Risk pathways for the introduction of the disease to your country
- ☐ NA
- ☐ other criteria (please describe) ______

21. Based on the latest prioritization, as answered in question 20, please list your top three **emerging** zoonoses from most important to least important for your **organization**. If you answered "NO" to question 20, please provide the three emerging zoonoses that you consider priority based on your experience. Please be as specific as possible and list genus, species, and subspecies if possible.

21.1 **Most** important **emerging** zoonosis

______

21.2 **Second** most important **emerging** zoonosis

______

21.3 **Third** most important **emerging** zoonosis

______

22. Either based on your experience, or from the latest formal prioritization conducted by your Ministry, please describe the probability of introduction to your country for the three top **emerging** zoonoses. If your Ministry did not elicit the probability of introduction, and you do not want to provide this information in your personal capacity, please select option “NA” below.

22.1 For the **most** important emerging zoonosis (as answered in question 21.1)

______

22.2 Please describe the time frame in which the probability in 22.1. applies.

______

22.3 For the **second** most important emerging zoonoses (as answered in question 21.2)

______

22.4 Please describe the time frame in which the probability in 22.3. applies.

______

22.5 For the **third** most important emerging zoonoses (as answered in question 21.3)

______

22.6 Please describe the time frame in which the probability in 22.5 applies.

______

23. Either based on your experience, or from the latest formal prioritization conducted by your Ministry please describe what impact/s were considered to rank the three top **emerging** zoonoses. If your Ministry did not elicit the impact, and you do not want to provide this information in your personal capacity, please select option “NA” below. Please select all options that apply.

23.1 For the **most** important emerging zoonosis (as in question 21.1)

- ☐ Impact on public health
- ☐ Impact on society
- ☐ Impact on economy
- ☐ Impact on environment
- ☐ Impact on public opinion
- ☐ NA
- ☐ Others (Please specify) ______

23.2 For the **second** most important emerging zoonosis (as in question 21.2)

- ☐ Impact on public health
- ☐ Impact on society
- ☐ Impact on economy
- ☐ Impact on environment
- ☐ Impact on public opinion
- ☐ NA
- ☐ Others (Please specify) ____

23.3 For the **third** most important emerging zoonosis (as in question 21.3)

- ☐ Impact on public health
- ☐ Impact on society
- ☐ Impact on economy
- ☐ Impact on environment
- ☐ Impact on public opinion
- ☐ NA
- ☐ Others (please specify) ____

24. Either based on your experience, or from the latest formal prioritization conducted by your Ministry please describe the **most important** risk pathway for the introduction to your country for the three top **emerging** zoonoses. If your Ministry did not elicit the risk pathways, and you do not want to provide this information in your personal capacity, please write “N/A”.

24.1 For the **most** important emerging zoonosis (as in question 21.1), please describe the most important risk pathway

____

24.2 For the **second** most important emerging zoonosis (as in question 21.2), please describe the most important risk pathway

____

24.3 For the **third** most important emerging zoonosis (as in question 21.3), please describe the most important risk pathway

____

25. Which **disease-specific** capacity (e.g. disease surveillance, laboratory diagnostic facilities, coordination of stakeholders) are you most interested in developing further in your country for your priority **emerging** zoonoses.

25.1 Top priority capacity to develop further for the **most** important emerging zoonoses (as in 21.1):

____

25.2 Top priority capacity to develop further for the **second** most important emerging zoonoses (as in 21.2):

____

25.3 Top priority capacity to develop further for the **third** most important emerging zoonoses (as in 21.3):

____

26. Do you have a current Memorandum of Understanding or other form of formal agreement with other government ministries (ie Ministry of Agriculture or Ministry of Health) for the coordinated prediction, prevention, detection and intervention of the three most important **emerging** zoonoses? (Select all that apply)

- ☐ For the top priority emerging zoonosis
- ☐ For the second priority emerging zoonosis
- ☐ For the third priority emerging zoonosis
- ☐ Other (please describe) [ ]

27. Do you have up-to-date contingency/emergency preparedness plans for the introduction of emerging zoonoses? Tick all that apply

- ☐ Yes, for the top priority emerging zoonosis
- ☐ Yes, for the second priority emerging zoonosis
- ☐ Yes, for the third priority emerging zoonosis

28. Have you conducted a simulation exercise of the introduction of emerging zoonoses in your country in the last five years? Tick all that apply.

- ☐ Yes, for the top priority emerging zoonoses
- ☐ Yes, for the second priority emerging zoonoses
- ☐ Yes, for the third priority emerging zoonoses

Section IV: General Section

29. Do you consider equity issues in your definition of priorities and allocation of resources against **emerging and/or endemic** zoonoses? That is, do you adjust the ranking of diseases for the fact that most zoonoses affects under-developed populations?

- ○ Yes
- ○ No
- ○ N/A

If yes, please describe below.

[ ]

30. Are you currently conducting syndromic surveillance targeted at earlier or more sensitive detection of any of the zoonoses **above**, either endemic or emerging? If yes, please indicate for which zoonoses below.

- ○ Yes
- ○ No

If yes, please indicate for which zoonotic diseases, emerging or endemic, you are conducting syndromic surveillance:

[ ]

31. How would you rank the relationship your ministry has with the Ministry of Health/Agriculture?

- ○ 1 - Very productive, we coordinate on many issues and share data
- ○ 2 - Overall productive with some coordinated activities and programs and some data sharing
- ○ 3 - Minimal productivity with few attempts to coordinate an activity or share data
- ○ 4 - No coordination between the ministries

32. How would you improve your coordination and collaboration with the Ministry of Health/Agriculture?

33. Specifically for the prediction, detection, prevention, and intervention of endemic and/or emerging zoonotic diseases, with who does your Ministry have a current formal agreement (e.g. a contract, Memorandum of Understanding, a written plan with clear roles and responsibilities)? Please select all that apply.

- ☐ i) Universities (e.g. to support technical advice)
- ☐ ii) NGOs (e.g. to support community-based interventions)
- ☐ iii) private sector
- ☐ iv) others (please describe)

34. What value would you allocate to a regular report (e.g. annually) about the occurrence of zoonoses in the Americas region, the status of capacities, etc. reflecting the information collected in this questionnaire? Please select one option.

- ○ Of great value
- ○ Of high value
- ○ Of some value
- ○ Of little value
- ○ Of no value

35. What other type of information would you like included in this report?

36. We are currently witnessing the transnational occurrence of a number of zoonotic conditions in the world, e.g. Chikungunya, MERS-CoV, Ebola and others. In your opinion, from a zoonoses programme perspective, what is **the most critical development** that t**he American region** requires to prevent the occurrence and spread of conditions like these? (e.g. better communication and collaboration between countries, formal network between countries, integrated surveillance between countries, etc)

Section V: Glossary

Administrative Staff: staff which assist in completing the organization's primary goals but have not completed education specific to these tasks; examples include secretaries, human resources, etc.

DALYs: disability-adjusted life year; the loss of one healthy year of life due to illness, disability, or premature mortality

Emerging: these are diseases which are not present in your country, also can be described as exotic

Endemic: these are diseases which are already present in your country

Equity: fairness or justice in the way people are treated

Memorandum of Understanding: formal, written agreement between organizations regarding coordination of activities; used here synonymously with national strategy, framework, action plan, mandate, agreement, etc.

Prioritize: to organize so that the most important thing is done or dealt with first

Risk Pathway: how a disease can or will enter the country or region

Technical Staff: staff trained in the technical aspects specific to the goals of the program; an example would be epidemiologists

Transnational: ability to affect multiple nations or move from nation to nation
